# Multiparametric Ultrasound Features of the Diffuse Sclerosing Variant of Papillary Thyroid Carcinoma: A Single-Center Case Series

**DOI:** 10.3390/diagnostics16020346

**Published:** 2026-01-21

**Authors:** Monica Latia, Stefania Bunceanu, Andreea Bena, Octavian Constantin Neagoe, Dana Stoian

**Affiliations:** 1 Department of Doctoral Studies, Victor Babeș University of Medicine and Pharmacy, 300041 Timișoara, Romania; monica.latia@umft.ro; 2 Dr. D Medical Center, Center for Advanced Ultrasound Evaluation, 300029 Timișoara, Romania; borlea.andreea@umft.ro (A.B.); stoian.dana@umft.ro (D.S.); 3 Center of Molecular Research in Nephrology and Vascular Disease, Faculty of Medicine, Victor Babeș University of Medicine and Pharmacy, 300041 Timișoara, Romania; 4 Second Department of Internal Medicine, Victor Babeș University of Medicine and Pharmacy, 300041 Timișoara, Romania; 5 Endocrinology Unit, Pius Brînzeu Emergency Clinical Hospital, 300723 Timișoara, Romania; 6 First Department of Surgery, Victor Babeș University of Medicine and Pharmacy, 300041 Timișoara, Romania; 7 Second Clinic of General Surgery and Surgical Oncology, Emergency Clinical Municipal Hospital, 300254 Timișoara, Romania

**Keywords:** diffuse sclerosing variant of papillary thyroid carcinoma, thyroid ultrasonography, shear-wave elastography, multiparametric ultrasound, cervical lymph node metastasis, autoimmune thyroiditis, preoperative assessment

## Abstract

**Background/Objectives:** The diffuse sclerosing variant of papillary thyroid carcinoma (DSV-PTC) is a rare and aggressive subtype characterized by diffuse gland involvement and early cervical lymph node metastasis. Preoperative differentiation from classic papillary thyroid carcinoma and autoimmune thyroid disease remains challenging on B-mode ultrasound. This study aimed to describe the multiparametric ultrasound features of DSV-PTC in a single-center case series and highlight practical imaging insights. **Methods:** We retrospectively reviewed seven consecutive patients with histologically confirmed DSV-PTC evaluated at a single center between 2013 and 2025. All patients underwent standardized B-mode ultrasound, color Doppler, and two-dimensional shear-wave elastography prior to surgery. Clinical, autoimmune, cytological, surgical, pathological, and follow-up data were analyzed descriptively. **Results:** The cohort included five females and two males (mean age 28 years). Autoimmune thyroid disease was present in three patients. High-risk ultrasound features were identified in all cases, with microcalcifications in six patients and a diffuse “snowstorm” appearance in five. Elastography demonstrated increased stiffness in six out of seven lesions (Emean 28–173 kPa; Emax 31–300 kPa). Cervical lymph node metastases were confirmed in all patients. In two cases, elastography aided identification of focal malignant involvement within diffusely altered thyroid parenchyma. All patients underwent total thyroidectomy with central neck dissection; lateral neck dissection and radioiodine therapy were performed selectively. No distant metastases were detected. **Conclusions:** In this case series, DSV-PTC showed a characteristic multiparametric ultrasound pattern combining high-risk B-mode features with frequently increased tissue stiffness. Elastography provided complementary information, particularly in the presence of autoimmune thyroid disease, by helping localize focal malignant involvement within diffusely altered parenchyma.

## 1. Introduction

Thyroid carcinoma is the most frequent endocrine malignancy, with papillary thyroid carcinoma (PTC) representing the predominant subtype [1]. Among its histopathologic variants, several (including the tall cell, hobnail, columnar cell, solid, and diffuse sclerosing variants) are associated with more aggressive clinical behavior [1,2]. The diffuse sclerosing variant of papillary thyroid carcinoma (DSV-PTC) is uncommon, accounting for approximately 0.7–5.3% of PTCs, and typically affects younger patients, often with a female predominance [2,3].

DSV-PTC presents unique diagnostic challenges. On conventional (B-mode) ultrasound (US), the gland is frequently diffusely enlarged and heterogeneous, sometimes closely resembling autoimmune thyroiditis [4]. However, DSV-PTC is pathologically characterized by widespread intrathyroidal involvement, extensive stromal fibrosis, psammoma body deposition, lymphovascular invasion, and dense lymphocytic infiltration, which are features associated with multifocality and early cervical lymph node metastasis [4,5,6]. The overlap between inflammatory changes related to autoimmune thyroid disease and malignant parenchymal involvement complicates accurate preoperative identification using B-mode US alone [4].

Shear-wave elastography (SWE) offers quantitative assessment of tissue stiffness and has shown promise in differentiating malignant from benign thyroid lesions. Case-based evidence suggests that DSV-PTC demonstrates markedly increased stiffness on SWE, reflecting its fibrotic and infiltrative tumor microenvironment [7]. More recently, combined diagnostic approaches incorporating US, elastography, and fine-needle aspiration cytology (FNAC) with optional molecular testing have been proposed to improve discrimination between DSV-PTC and autoimmune thyroiditis [5].

The objective of this study was to characterize the preoperative multiparametric US features of DSV-PTC in a single-center cohort and to describe their relationship with cytologic assessment, cervical lymph node involvement, and postoperative pathological outcomes.

While previous studies have reported individual sonographic features of DSV-PTC or isolated elastography findings, most consist of single case reports or small series without standardized multiparametric imaging or comprehensive imaging–pathology correlation [4,7,8]. The present study expands on the existing literature by providing a systematically acquired, multiparametric US characterization of DSV-PTC, integrating B-mode US, Doppler assessment, and SWE with cytologic, surgical, and histopathologic correlation, with particular attention given to diagnostic challenges posed by coexisting autoimmune thyroid disease.

## 2. Materials and Methods

### 2.1. Study Design and Patients

This study was designed as a retrospective descriptive case series, aiming to characterize imaging patterns rather than to test diagnostic performance. It included seven consecutive patients diagnosed with DSV-PTC who underwent preoperative US evaluation, including two-dimensional shear-wave elastography (2D-SWE), at Dr. D Medical Center in Timișoara, Romania, between January 2013 and June 2025, spanning a total of 12.5 years. During this period, a total of 470 thyroid cancers were diagnosed at our center, comprising 425 PTCs, of which 128 were micro-PTC and 7 DSV-PTC, 25 follicular thyroid carcinomas (FTC), and 20 medullary thyroid carcinomas (MTC).

**Figure 1:**
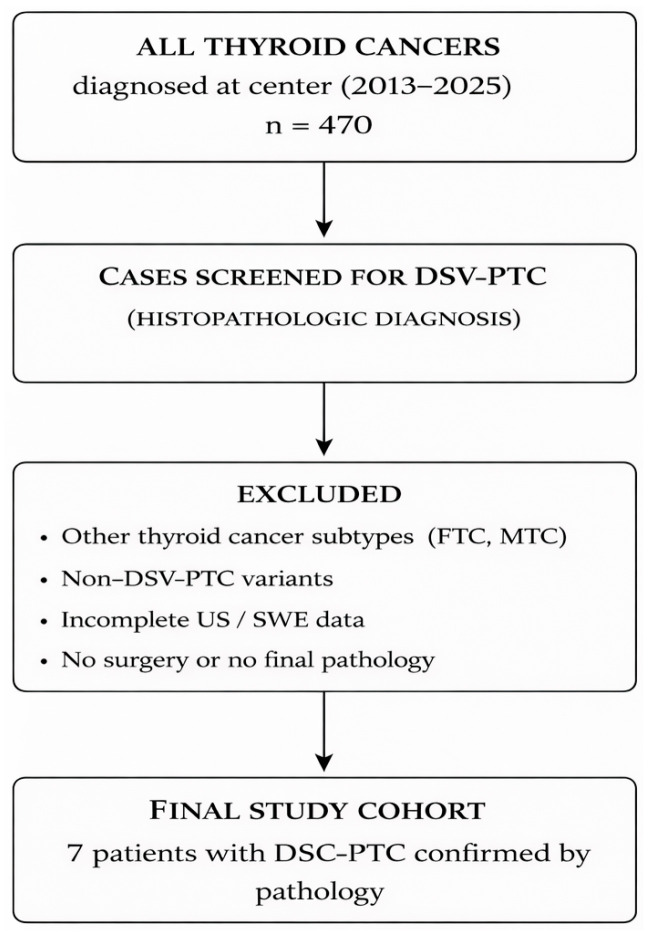
Flowchart of patient selection for the study cohort.

Inclusion criteria were: surgical treatment with subsequent histological diagnosis of DSV-PTC, availability of preoperative FNAC results from the dominant thyroid lesion and/or suspicious lymph nodes, availability of complete preoperative B-mode and color Doppler US examinations, and availability of qualitative and quantitative 2D-SWE measurements. Patients were excluded if they were diagnosed with a different thyroid cancer subtype, lacked complete US or elastography data, were evaluated in another institution, did not undergo surgery, or had no final pathology report available. Patient selection and inclusion are summarized in Figure 1.

The study was approved by the Ethics Committee of Victor Babeș University of Medicine and Pharmacy, Timișoara (Approval No. 52/2 October 2023), and conducted in accordance with the Declaration of Helsinki. Written informed consent for the use of clinical data and anonymized imaging for research and publication was obtained from all participants; all data were fully anonymized prior to analysis, with no patient identifiers retained.

### 2.2. Clinical and Laboratory Data

For each patient, demographic and clinical data were collected, including age, sex, presenting symptoms, serum thyroid-stimulating hormone (TSH) and free thyroxine (FT4) levels, and the presence of autoimmune thyroid disease, assessed by anti-thyroid peroxidase antibodies (anti-TPO) and anti-thyroglobulin antibodies (anti-Tg). Past thyroid disease history and prior exposure to neck irradiation, chemotherapy, or hematologic malignancy were documented when applicable. Surgical and histopathological reports were reviewed to record tumor focality (unifocal vs. multifocal), presence of extrathyroidal extension, lymphovascular invasion, and the number and distribution of lymph nodes dissected.

### 2.3. B-Mode US and 2D-SWE Protocol

All examinations were performed by a single operator with over 15 years of experience in thyroid ultrasonography, using either the Aixplorer Mach 30 system (SuperSonic Imagine, Aix-en-Provence, France) equipped with an L18-5 linear probe (5–18 MHz) or the Aixplorer system (SuperSonic Imagine, Aix-en-Provence, France) with a high-frequency SL15-4 linear probe (4–15 MHz) [9]. Patients were positioned supine with the neck slightly hyperextended to optimize anterior cervical exposure, supported by a small pillow for stabilization. A generous amount of coupling gel was applied, and minimal probe pressure was used to avoid tissue compression artifacts. B-mode assessment included thyroid volume, echogenicity (compared to the submandibular gland), homogeneity, and the presence of diffuse or focal hypoechoic areas and microcalcifications. We defined a “snowstorm” pattern as diffusely distributed punctate echogenic foci ≤ 1 mm without posterior acoustic shadowing [8]. Vascularity was assessed on color Doppler and recorded as normal or increased.

When focal nodules were present, standard features were recorded (location, maximum diameter, shape, margins, composition, echogenicity, presence of hyperechoic foci/calcifications, and vascularity), following established suspicious US criteria [10]. Lateral neck compartments were scanned and suspicious lymph nodes were defined as lacking a fatty hilum, showing microcalcifications, cortical hyperechogenicity, cystic change, or irregular vascularity [11].

Subsequently, 2D-SWE was performed in longitudinal planes using ultrafast imaging technology that generates supersonic shear waves, enabling real-time visualization of tissue elasticity. The resulting elastograms were displayed as color-coded maps, where blue indicated soft tissue and red represented stiffer areas [12,13]. To ensure reliable measurements, the transducer was held steady and perpendicular to the skin with minimal pressure, and patients were instructed to avoid swallowing or moving during image acquisition [14]. The acquisition box was adjusted to fully encompass the nodule and optimize elastogram quality.

For quantitative analysis, at least three to five valid measurements were obtained for each imaged region, with a 2–4 mm circular region of interest (ROI) placed over the most suspicious focal lesion within the thyroid parenchyma (dominant nodule or focal stiff area when no discrete nodule was present), and on suspicious cervical lymph nodes when applicable, carefully avoiding calcifications, cystic zones, and artifacts. The mean (Emean), minimum (Emin), and maximum (Emax) elasticity indices (EIs) were recorded in kilopascals (kPa). The QBox ratio, defined as the ratio between the elasticity of the lesion and that of adjacent normal parenchyma or muscle, was also calculated when applicable [12]. Imaging presets, ROI size, and acquisition depth were standardized across all patients. A qualitative assessment was also performed using an elasticity score (ES), categorizing nodules into four color-coded patterns: ES 1 (homogeneous, elastic, completely blue); ES 2 (blue with vertical green stiffness lines); ES 3 (eccentric stiffness); ES 4 (central stiffness with heterogeneity) [15]. Nodules classified as ES 3 or 4 were considered high-risk. The qualitative elasticity score (ES 1–4) was used as a visual adjunct to quantitative SWE measurements, based on increasing stiffness patterns described in prior thyroid elastography studies [15]. All SWE measurements were performed by the same experienced operator to minimize interobserver variability.

In DSV-PTC, increased stiffness is frequently observed not only within focal nodular lesions but also diffusely throughout the affected parenchyma, reflecting the extensive fibrosis and inflammatory infiltration characteristic of this subtype. This pattern was considered when interpreting the elastograms, as generalized stiffness may coexist with focal regions of higher elasticity contrast corresponding to dominant tumor foci [5,7,8].

### 2.4. FNAC and Pathology

FNAC was performed using a 25–27G needle (B. Braun, Melsungen, Germany) under US guidance; at least two passes were obtained per lesion/region, and cytology was reported according to the Bethesda System (I–VI) [16]. Surgical specimens were processed routinely with hematoxylin and eosin staining. An experienced endocrine pathologist confirmed DSV-PTC based on established histopathological criteria (diffuse involvement, abundant psammoma bodies, squamous metaplasia, extensive lymphocytic infiltration, and stromal fibrosis). Final tumor staging was assigned according to the 8th edition of the American Joint Committee on Cancer (AJCC) TNM classification system [6].

### 2.5. Data Analysis

This study is descriptive in nature. Categorical variables are reported as counts and percentages, while continuous variables are summarized descriptively (mean/range or median/range). Individual case-level information, including demographic characteristics, US features, SWE measurements (mean, minimum, and maximum), FNAC classification, and surgical pathology findings, is presented in structured summary tables. No inferential statistical testing was performed. Data organization and descriptive analysis were conducted using Microsoft Excel (Microsoft Corporation, Redmond, WA, USA, version 365).

## 3. Results

The study included seven patients (five females and two males), with a mean age of 28 years (range 17–49 years) (Table 1). A family history of thyroid disease or autoimmunity was identified in three cases (43%), and two patients (29%) had a history of prior irradiation or chemotherapy during childhood or adolescence (one for acute lymphoblastic leukemia and one for Hodgkin lymphoma treated with total body irradiation and stem cell transplantation). Evidence of autoimmune thyroiditis, defined by elevated anti-TPO and/or anti-Tg, was observed in three patients (43%). Baseline thyroid function was euthyroid in all cases (median TSH 1.84 µIU/mL, range 1.18–3.72; mean FT4 16.7 pmol/L, range 15.7–18). One patient (Case 5) had a history of Graves’ disease (TRAb-positive) and was evaluated after achieving euthyroidism during antithyroid treatment.

**Figure 2:**
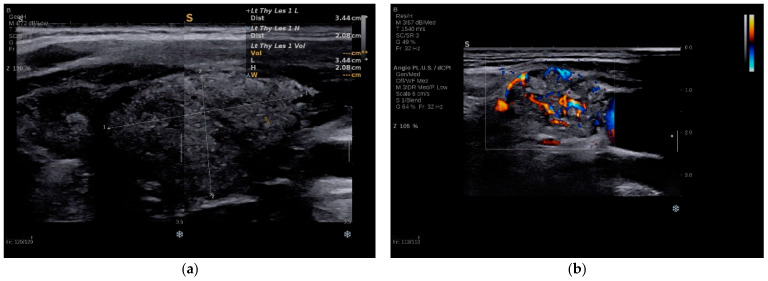
B-mode US evaluation of Case 1. (**a**) Heterogeneous, markedly hypoechoic left lobe with diffuse punctate echogenic foci (“snowstorm” pattern); (**b**) Suspicious lateral cervical lymph node with loss of hilar architecture, microcalcifications and increased vascularity on color Doppler.

All patients had high-suspicion thyroid nodules, classified as TIRADS 5 on B-mode assessment (Table 1). Nodules were solid and markedly hypoechoic, commonly with ill-defined or irregular margins. Microcalcifications were identified in six of seven patients (86%), with five cases exhibiting the typical “snowstorm” appearance (71%), characterized by numerous punctate echogenic foci throughout the affected parenchyma. Most lesions were located in the mid-to-lower pole of the thyroid lobes, while their size ranged from 0.8 to 2.9 cm (median 1.6 cm). Two cases (Cases 5 and 6) demonstrated bilateral high-risk nodules (29%), while the remaining patients had solitary or dominant nodules (71%). Color Doppler demonstrated increased intranodular vascularity in all cases, with chaotic intranodular flow patterns in five cases (71%). Suspicious cervical lymph nodes were detected on preoperative US in four patients (47%), most commonly involving central compartment (level VI) nodes, with or without involvement of lateral levels III–IV. In Case 1, a metastatic retrojugular lymph node was first detected on contrast-enhanced chest CT, prompting directed cervical evaluation. Representative B-mode US images from Case 1 are shown in Figure 2.

**Figure 3:**
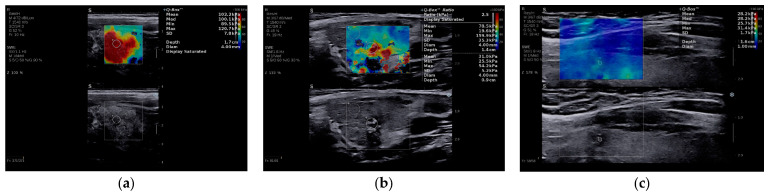
Representative SWE patterns in DSV-PTC. (**a**) Case 1: diffusely heterogeneous thyroid parenchyma without a clearly defined dominant nodule on B-mode US; SWE demonstrates a focal area of markedly increased stiffness, aiding lesion localization; (**b**) Case 5: hypoechoic thyroid nodule with corresponding high stiffness on SWE; (**c**) Case 4: thyroid nodule with low stiffness values on SWE, illustrating a potential false-negative elastographic pattern.

**Figure 4:**
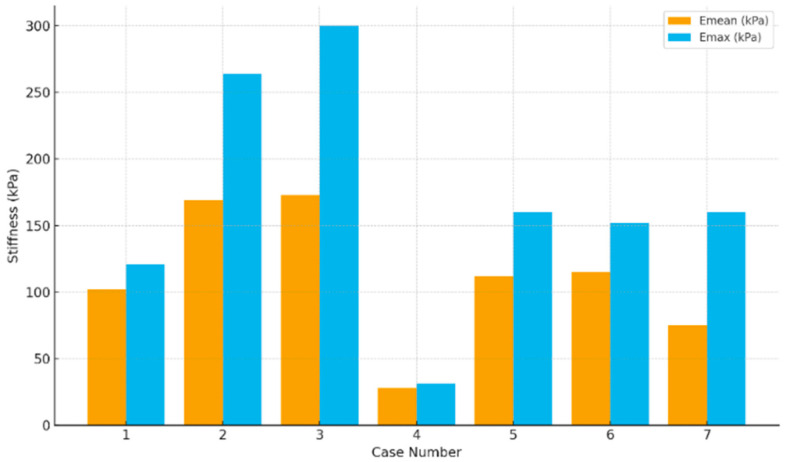
Comparison of Emean and Emax across the seven cases.

SWE was performed in all seven cases (Table 1). The qualitative ES averaged 3.7 (range 2–4), and six of seven nodules (86%) demonstrated marked stiffness (ES 4), with Case 4 being the only lesion demonstrating lower stiffness (ES 2). This pattern was consistent with diffuse stromal fibrosis characteristic of DSV-PTC, rather than size or vascularity alone. On quantitative assessment, Emean values ranged from 28 to 173 kPa (mean 110 kPa), while Emax ranged from 31 to 300 kPa. The lowest stiffness was observed in Case 4 (Emean 28 kPa, Emax 31 kPa), whereas Cases 2 and 3 demonstrated the highest stiffness, with Emean ≥ 160 kPa and Emax reaching 300 kPa. Figure 3 illustrates representative elastographic patterns, including cases in which SWE aided lesion localization in diffusely altered parenchyma, typical high-stiffness nodules, and a low-stiffness outlier. Figure 4 displays the comparative distribution of Emean and Emax values across the cohort.

**Figure 5:**
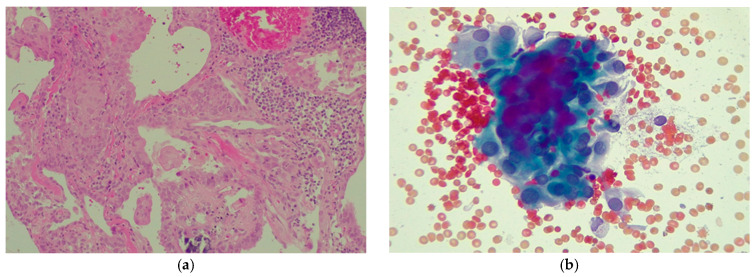
Representative imaging and pathologic findings in Case 1. (**a**) Core needle biopsy histology of the thyroid lesion (hematoxylin–eosin staining); (**b**) Cytology of a metastatic lymph node (Papanicolaou staining).

**Figure 6:**
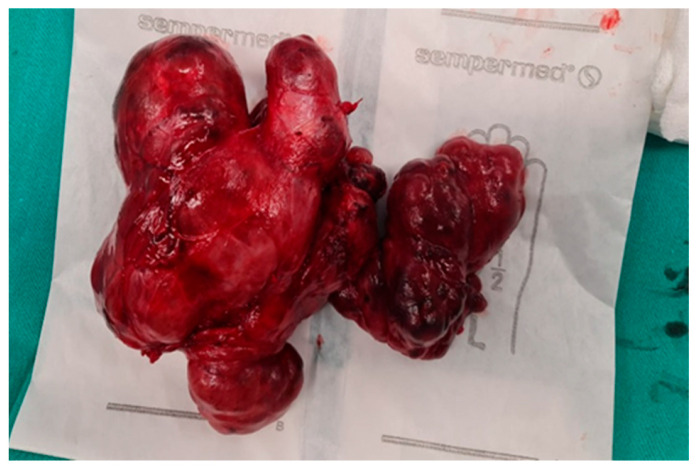
Representative surgical findings in Case 1 showing the excised thyroid gland with attached nodal tissue.

Lymph node involvement was confirmed in all seven patients (100%) (Table 2). Preoperative FNAC was performed in all cases, yielding Bethesda category V or VI cytology in the dominant thyroid lesions and/or suspicious lymph nodes, supporting the preoperative suspicion of malignancy. The total number of excised lymph nodes ranged from 3 to 63 per patient, with 1 to 32 metastatic nodes identified. Metastatic involvement most frequently affected the central compartment (level VI), while lateral cervical involvement (levels III–IV) was present in cases with more extensive regional disease, particularly Cases 1 and 7. Although histopathology confirmed cervical lymph node metastases in all patients, preoperative US identified suspicious lymph nodes in only four of seven cases, highlighting a discordance between imaging and pathological findings. This finding underscores the infiltrative and multifocal behavior of DSV-PTC and supports the need for comprehensive preoperative assessment and careful surgical planning. Multifocal carcinoma was documented in four cases, and all specimens demonstrated histopathological features characteristic of DSV-PTC, including dense stromal fibrosis, prominent lymphocytic infiltration, and widespread psammoma bodies. Figure 5 and Figure 6 present representative imaging, pathologic, and surgical findings from Case 1. Cases with higher stiffness values demonstrated a greater number of metastatic nodes, while cases with lower stiffness values were associated with fewer involved nodes. This observation is descriptive in nature and reflects a qualitative trend within the cohort; no statistical testing was performed due to the small sample size. In clinical terms, stiffness patterns aided preoperative suspicion of nodal disease, supporting compartment-oriented surgical planning in cases with high SWE values.

All patients underwent total thyroidectomy, and five of seven subsequently received radioiodine (RAI) therapy (Table 2). RAI activity ranged from 3.07 to 14.97 GBq and was individualized according to nodal burden and biochemical response. One patient required a single RAI course (Case 2), while four required multiple treatments for persistent cervical disease. Two patients (Cases 3 and 4) did not complete postoperative RAI therapy due to loss to follow-up after surgery. Postoperative thyroglobulin (Tg) was low or undetectable in three patients (<0.04–0.479 ng/mL), while anti-Tg levels varied in relation to autoimmune thyroiditis. Case 6, previously treated with mediastinal irradiation and requiring multiple RAI treatments (total 14.97 GBq), demonstrated fluctuating Tg levels but stable cervical findings under TSH suppression. Among patients who completed follow-up (five of seven patients; median 30 months, range 14–96), no distant metastases were detected on imaging. Two patients achieved an excellent response at the last evaluation, while three (Cases 1, 5, and 6) remained under active surveillance due to stable minimal residual nodal disease and/or detectable Tg. All patients remained on TSH-suppressive levothyroxine, with no major treatment-related adverse effects observed.

## 4. Discussion

DSV-PTC is an uncommon but clinically aggressive subtype characterized by diffuse intraglandular spread and early cervical lymph node metastases. During the study period, 470 thyroid cancers were diagnosed in our center, including 425 PTC, of which seven were DSV-PTC (1.65%), aligning with the reported rarity of this subtype (0.7–5.3%) [1,2,17,18]. In our cohort, patients were predominantly young and female, and all presented with nodal involvement at diagnosis, while four exhibited multifocal intrathyroidal disease, consistent with previously reported clinical and pathologic features of DSV-PTC [19,20]. Notably, two patients had a history of childhood or adolescent therapeutic radiation exposure, consistent with the recognized association between prior irradiation and later PTC development [21]. Autoimmune thyroid disease was present in half of the cases, including patients with autoimmune thyroiditis and one patient with Graves’ disease, a coexistence previously linked to both parenchymal heterogeneity and cytologic interpretive challenges [22].

On B-mode evaluation, DSV-PTC demonstrated a heterogeneous spectrum of findings in our cohort. All patients exhibited high-suspicion features, including marked hypoechogenicity, ill-defined or irregular margins, and diffuse or focal microcalcifications. The characteristic “snowstorm” appearance, defined by diffusely distributed punctate echogenic foci suggestive of psammoma bodies, was observed in a majority of cases but was not universal, reflecting the variable imaging presentation of this subtype [8,23]. In several patients, the thyroid gland appeared diffusely enlarged and heterogeneous, with poorly defined focal lesions blending into the surrounding parenchyma, particularly in the presence of coexisting autoimmune thyroid disease. Dense lymphocytic infiltration, a hallmark of both DSV-PTC and autoimmune thyroiditis, further contributed to parenchymal heterogeneity and cytologic overlap on FNAC, representing a well-recognized diagnostic pitfall [24]. These B-mode features underscore the difficulty of confidently identifying dominant malignant foci within diffusely altered glands using conventional imaging alone and may contribute to underestimation of disease extent on preoperative US in DSV-PTC [4,8].

In all patients, SWE added complementary information to B-mode assessment by characterizing tissue stiffness. Quantitatively, stiffness values were generally high, with Emean ranging from 28 to 173 kPa and Emax from 31 to 300 kPa. On qualitative assessment, six of seven nodules (86%) were ES 4, consistent with markedly increased rigidity. However, stiffness was not uniform across lesions: several cases showed heterogeneous SWE maps, likely reflecting the patchy distribution of stromal fibrosis, lymphocytic infiltration, and psammoma body deposition that characterizes DSV-PTC. Similar heterogeneous patterns, with alternating stiffer and less stiff areas corresponding to microscopic fibrosis distribution, have been reported in prior DSV-PTC series, supporting the concept that SWE may capture tumor microstructure rather than size alone [5,7,8,23]. Notably, Case 4 was an outlier, with unexpectedly low SWE values despite histologically confirmed DSV-PTC. This may reflect limited fibrosis/desmoplastic response or early-stage disease and illustrates a potential false-negative elastography scenario. Accordingly, low stiffness should not be used to exclude DSV-PTC, and SWE findings should be interpreted in conjunction with the overall US pattern and clinical context [25,26].

While FNAC remains the standard initial diagnostic test for thyroid nodules, SWE provided valuable complementary information in our series, particularly when biopsy was technically limited by dense fibrosis or calcification. In Case 1, marked sclerosis and coarse microcalcifications reduced cytologic cellularity on FNAC, necessitating core biopsy for definitive diagnosis; however, SWE had already demonstrated markedly increased stiffness, supporting suspicion of an infiltrative malignant process and facilitating timely surgical management. Similar reports indicate that SWE may aid clinical decision-making in nodules with nondiagnostic or indeterminate cytology, especially when fibrosis or calcified foci impair aspiration [27,28]. Nevertheless, increased stiffness is not specific to malignancy and may also be observed in advanced autoimmune thyroiditis [29]. Accordingly, SWE should not replace histologic evaluation but may assist in selecting targets for repeat sampling, identifying the stiffest regions for biopsy, and supporting preoperative risk assessment when interpreted alongside B-mode US, cytology, and clinical context.

Distinguishing DSV-PTC from classic PTC and autoimmune thyroiditis on B-mode US alone is challenging, as all three entities may present with diffuse hypoechogenicity and heterogeneous parenchyma [5,30]. In two patients in our cohort (Cases 1 and 7), the dominant malignant lesions were initially interpreted as diffuse parenchymal changes rather than discrete nodules because of marked heterogeneity and poorly defined margins characteristic of autoimmune thyroiditis. In these cases, high-resolution US combined with SWE revealed focal areas of stiffness markedly exceeding that of the surrounding parenchyma, enabling targeted FNAC and subsequent confirmation of DSV-PTC. These observations illustrate a well-recognized diagnostic challenge in DSV-PTC, in which malignant infiltration may be obscured within a diffusely altered gland, and support the incremental value of elastography in identifying malignant foci that might otherwise be overlooked on B-mode imaging alone [4,7,8].

In our cohort, the addition of SWE improved discriminatory confidence by highlighting stiffness patterns that differed from those typically observed in classic PTC or autoimmune thyroiditis. DSV-PTC lesions generally demonstrated higher stiffness values, whereas autoimmune thyroiditis more often produced diffuse mild-to-moderate stiffness without discrete regions of marked rigidity [31,32]. In glands diffusely altered by autoimmune thyroid disease, focal stiffness disproportionate to the surrounding parenchyma may therefore raise suspicion for infiltrative carcinoma. This pattern is consistent with the histologic features of DSV-PTC and with previously published elastography reports, including cases showing substantial overlap with autoimmune thyroiditis but overall higher stiffness in DSV-PTC [5,7]. Importantly, major elastography guidance documents do not propose DSV-PTC-specific SWE cut-off values. Both WFUMB and EFSUMB guidelines outline technique, interpretation, and known pitfalls of thyroid elastography, but emphasize that variant-specific thresholds cannot be recommended because of limited evidence [33,34]. Consequently, stiffness cut-offs validated for general thyroid nodules should not be applied directly to DSV-PTC. Instead, SWE should be used as an adjunct to B-mode US and cytology, with careful attention to technique and region-of-interest placement away from coarse calcifications to minimize stiffness overestimation [33,34].

Cervical lymph node metastasis was confirmed in all seven patients in this cohort, consistent with the well-documented propensity of DSV-PTC for early and multifocal lymphatic spread [35,36]. Metastatic involvement most frequently affected the central compartment, while lateral neck dissemination was observed in cases with more extensive regional disease. When examining the relationship between tumor stiffness and nodal burden, a qualitative trend emerged: cases with markedly elevated SWE stiffness, either high Emean values (≥150 kPa) or very high Emax values (≥250–300 kPa), tended to demonstrate more extensive metastatic involvement. In contrast, Case 4, which exhibited low stiffness (Emean 28 kPa), was associated with only micrometastatic nodal disease. These observations are consistent with prior studies suggesting that stromal fibrosis, lymphocytic infiltration, and psammoma body accumulation, hallmarks of DSV-PTC, contribute both to increased tissue stiffness and to aggressive lymphatic dissemination [8,37]. However, this association was not deterministic. Cases with intermediate stiffness values also harbored metastatic lymph nodes, indicating that SWE likely reflects tumor stromal biology rather than metastatic burden alone. Although a positive trend between Emean values and the number of metastatic nodes was observed, this finding is purely descriptive, and the small sample size precludes statistical analysis or formal predictive modeling. Taken together, these findings support the use of SWE as a contextual risk marker that may aid preoperative assessment and nodal mapping, rather than as a stand-alone predictor of metastatic spread [38].

All patients underwent total thyroidectomy with central neck dissection, reflecting the recognized tendency of DSV-PTC for early central compartment lymphatic spread. Lateral neck dissection was performed selectively when metastatic involvement was confirmed on preoperative US or cytology [39]. This surgical approach is consistent with current American Thyroid Association 2025 guideline principles, which emphasize compartment-oriented neck dissection when therapeutic lymph node dissection is indicated, particularly in tumors with aggressive behavior and a high nodal metastatic burden [40]. In this cohort, multiparametric US findings, including B-mode imaging, cervical lymph node assessment, and SWE, contributed not only to diagnostic evaluation but also to preoperative risk stratification and surgical planning. By improving delineation of disease extent and nodal involvement, imaging supported more informed decisions regarding the scope of surgery and reinforces the role of advanced US techniques in precision-oriented management of DSV-PTC.

A key strength of this study lies in its standardized multiparametric US assessment, including B-mode imaging, color Doppler evaluation, and SWE, performed by a single experienced operator using the same US platform. This methodological consistency ensured uniform ROI placement, acquisition depth, and measurement technique, allowing reliable intra-cohort comparison of stiffness patterns. Unlike prior reports that are largely limited to single cases or isolated imaging modalities, this study integrates US findings with detailed cytologic, surgical, and histopathologic correlation, thereby providing clinically meaningful insight into how stromal fibrosis, psammoma body deposition, and lymphocytic infiltration translate into characteristic imaging patterns in DSV-PTC. In addition, the inclusion of patients with coexisting autoimmune thyroid disease highlights a frequent real-world diagnostic confounder that has been underrepresented in previous imaging studies of this rare variant.

Several limitations should be acknowledged. First, the small sample size reflects the rarity of DSV-PTC and limits statistical inference, precluding the definition of subtype-specific SWE cut-off values. However, for rare entities such as DSV-PTC, large prospective imaging cohorts are difficult to assemble, and detailed single-center case series with standardized acquisition and imaging–pathology correlation represent an appropriate and necessary exploratory approach. In this context, the present study is intended to be hypothesis-generating rather than validating. Second, the retrospective design may introduce selection bias toward patients with more advanced or clinically apparent nodal disease. Third, although SWE measurements were internally consistent, inter-observer and inter-platform reproducibility was not assessed, and findings may not be directly generalizable across elastography systems. Finally, postoperative follow-up was not uniform across the cohort, as one patient did not return for scheduled evaluation and another had not yet completed initial RAI therapy, limiting consistent long-term outcome assessment. Larger prospective, multicenter studies with standardized elastography protocols will be required to further clarify the diagnostic and prognostic utility of stiffness metrics in DSV-PTC and to define how elastography may best be integrated into preoperative risk stratification and surgical decision-making.

## 5. Conclusions

This case series describes the characteristic clinical and multiparametric US features observed in patients with DSV-PTC. In all cases, B-mode US demonstrated high-risk findings, while SWE revealed increased tissue stiffness in most patients, consistent with the fibrotic and calcified tumor microenvironment typical of this variant. In two patients with coexisting autoimmune thyroid disease, extensive parenchymal heterogeneity initially obscured the presence of a discrete tumor on B-mode imaging; in these cases, the addition of SWE helped localize focal areas of increased stiffness and guided targeted diagnostic FNAC. Although stiffness values varied among individuals, markedly elevated SWE measurements were more often observed in cases with greater nodal involvement, suggesting that elastography may serve as a contextual indicator of tumor stromal characteristics rather than as a stand-alone marker of aggressiveness. Cervical lymph node metastases were present in all patients, underscoring the importance of thorough preoperative nodal assessment and compartment-oriented surgical planning in DSV-PTC. Despite the high prevalence of nodal disease at presentation, most patients demonstrated favorable biochemical and structural outcomes following total thyroidectomy, risk-adapted RAI therapy, and TSH suppression.

Taken together, these observations suggest that SWE may provide complementary information when integrated into a multiparametric US approach for the evaluation of suspected DSV-PTC, particularly in cases where B-mode findings overlap with autoimmune thyroid disease. Future research should focus on prospective, multicenter studies using standardized SWE acquisition and reporting protocols to better define reproducible stiffness patterns, assess inter-platform comparability, and clarify the role of elastography in preoperative risk stratification, surgical planning, and long-term follow-up.

## Figures and Tables

**Table 1:** Clinical and multiparametric US characteristics of the seven patients with DSV-PTC.

Case	Age/Sex	Family/Personal History	Thyroiditis (ATPO/ATG)	TSH (µIU/mL)	FT4 (pmol/L)	Nodule Location	Nodule Size (cm)	B-Mode US Aspect & TIRADS	Suspicious Lymph Nodes on US	SWE (Emean/Emax, kPa)
1	23/M	Autoimmune thyroid disease (2023), no LT4; no FHx	ATPO 110; ATG 80	3.6	17.4	Left lobe	2.5	Enlarged gland; L hypoechoic, heterogeneous with “snowstorm” foci, chaotic Doppler; TIRADS 5	Medial jugular; bilateral lateral & supraclavicular	Nodule: 102/121; Lymph node: 280/300 (depth 1.5–1.7 cm, ROI 4 mm)
2	49/F	Autoimmune thyroid disease (2014), LT4 87.5 µg/day; no FHx	ATPO 75; ATG < 1.3 (negative)	3.72	17.6	Left lobe	1.1	Reduced volume; R heterogeneous ill-defined with echogenic foci; L hypoechoic sclerosing appearance with “snowstorm” foci; chaotic Doppler (bilateral); TIRADS 5	None reported	169/264 (depth 1.2 cm, ROI 4 mm)
3	24/F	No significant PMH; FHx autoimmune thyroid disease	ATPO < 1; ATG < 1.3 (negative)	1.9	16.5	Left lobe	2.2	Solid, hypoechoic, heterogeneous with “snowstorm” foci, chaotic Doppler; TIRADS 5	Inferior left level VI	173/300 (depth 1.3 cm, ROI 4 mm)
4	37/M	No significant PMH; FHx thyroid disease (unspecified)	ATPO < 1; ATG < 1.3 (negative)	1.48	16	Left lobe	0.8	Solid, intensely hypoechoic, ill-defined; TIRADS 5	None reported	28/31 (depth 1.6 cm, ROI 2 mm)
5	19/F	Childhood ALL (chemo + cervical/thoracic RT); Graves’ disease at 19; FHx multiple neoplasia and autoimmune thyroid disease	(thyroiditis not primary issue; TRAb 9.7 IU/L)	1.2	18	Bilateral	L: 0.7; R: 2.9	L: intensely hypoechoic, taller-than-wide, “snowstorm” foci, chaotic Doppler; R: hypoechoic, ill-defined, inhomogeneous, microcalcifications; TIRADS 5 (bilateral)	Bilateral level VI, right lateral levels III and IV	L: 78.5/160 (depth 1.4 cm, ROI 4 mm); R: 112/152 (depth 1.2 cm, ROI 4 mm)
6	26/F	Hodgkin lymphoma at 18 (chemo, stem-cell transplant, whole-body RT); congenital thrombophilia; no FHx	ATPO < 1; ATG < 1.3 (negative)	1.84	15.9	Bilateral	L: 1.2; R: 1	Marked hypoechoic, irregular, taller-than-wide, microcalcifications; TIRADS 5 (bilateral)	None reported	L: 115/152 (depth 1.2 cm, ROI 2 mm); R: 65/108 (depth 1.9 cm, ROI 2 mm)
7	17/F	Autoimmune thyroiditis since age 9, no LT4; no FHx	ATPO 14.8; ATG 755	1.18	15.70	Left lobe	1.9	Hypoechoic, inhomogeneous, irregular margins, “snowstorm” foci, chaotic Doppler; TIRADS 5	Multiple left nodes (levels III–IV)	75/160 (depth 1.8 cm, ROI 4 mm)

PMH, personal medical history; FHx, family history; LT4, levothyroxine; TIRADS, Thyroid Imaging Reporting and Data System; US, ultrasound; ATPO, anti-thyroid peroxidase antibodies; ATG, anti-thyroglobulin antibodies; TSH, thyroid-stimulating hormone; FT4, free thyroxine; TRAb, TSH receptor antibodies; SWE, shear-wave elastography; ALL, acute lymphoblastic leukemia; RT, radiotherapy.

**Table 2:** Other imaging, cytology, pathology, staging, treatment, and follow-up outcomes in the seven DSV-PTC patients.

Case	Other Imaging	FNAC (Bethesda)	Pathology Result	TNM (AJCC 8th)	LNs Excised (Met/Total)	Postop Tg/ATG	RAI (GBq)	Follow-up Duration (Months)/Outcome
1	CEUS (late wash-in/early wash-out); serial contrast chest CT; PET-CT (bilateral cervical/supraclavicular uptake); brain MRI negative	Core needle biopsy thyroid: PTC features; FNAB lymph node: PTC cytology	DSV-PTC; multifocal; Hashimoto background; perineural invasion; pericapsular fat infiltration	pT4(m)N1b	32/63	Stimulated Tg 0.23 ng/mL after 2nd RAI; ATG fell to below limit	3.07 + 3.69	14 months/Re-intervention for right retrojugular LN metastasis; on TSH-suppressive therapy (TSH < 0.1 mIU/L); whole-body scan negative; ongoing surveillance with planned US and Tg assessment.
2	None needed preop	VI	DSV-PTC with microvascular invasion; Hashimoto background (L)	pT1bN1	1/3	Stimulated Tg < 0.04 post-RAI; latest 0.18 at 3 years	3.7	42 months/On appropriate TSH suppression; no evidence of local or distant recurrence during follow-up.
3	None	VI (nodule & LNs)	DSV-PTC	pT2N1a	4/4	—	Indicated, not confirmed received	138 months/Lost to follow-up after surgery; patient non-compliant and has not presented for formal postoperative evaluation.
4	None	V	Unencapsulated DSV micro-PTC; peritumoral lymphatic invasion	pT1aN1	1/3	Stimulated Tg 0.479 ng/mL; ATG < 1.3	Pending	114 months/On TSH suppression; referred for RAI therapy but has not returned for post-RAI follow-up evaluation.
5	None	VI	DSV-PTC; multifocal bilateral	pT1bN1	4/6	2 months postop: stimulated Tg 56.9 ng/mL, ATG 13.7 → later suppressed Tg 6.6 ng/mL	3.36 + 1.20 (third planned)	20 months/Persistent central and lateral cervical adenopathy on US despite two RAI treatments; additional RAI therapy planned.
6	PET-CT (retrosternal mass); MRI later for follow-up	VI	DSV-PTC; left index; multifocal right	pT1b(m)N1a L1 V R0	4/6	Initial stimulated Tg 13.81 ng/mL; ATG < 1.3	2.197 + 2.59 + 4.006 + 3.076 + 3.07 + 3.07 (total 14.97)	96 months/On TSH suppression; variable Tg levels; WBS, MRI, and PET imaging negative for distant disease; fluctuating cervical US findings; ongoing follow-up.
7	PET-CT at follow-up	V	DSV-PTC; multifocal bilateral	pT1b(m)N1b L1 V0 Pn0 R0	11/38	Early high ATG 228 → 64; stimulated Tg < 0.04 at 34 months	3.07 + 3.07 + 8.73	30 months/No evidence of disease on US and PET-CT following reoperation and third RAI course; excellent response; on TSH suppression.

FNAC, fine-needle aspiration cytology; TNM, tumor node metastasis staging system; LN, lymph node; Tg, thyroglobulin; ATG, anti-thyroglobulin antibodies; RAI, radioiodine therapy; CEUS, contrast-enhanced ultrasound; PET-CT, positron emission tomography-computed tomography; MRI, magnetic resonance imaging; FNAB, fine-needle aspiration biopsy; WBS, whole-body scintigraphy.

## Data Availability

The data presented in this study are available on request from the corresponding author due to privacy and ethical restrictions related to patient data.

## Abbreviations

The following abbreviations are used in this manuscript:

DSV-PTC	diffuse sclerosing variant of papillary thyroid carcinoma
PTC	papillary thyroid carcinoma
FTC	follicular thyroid carcinoma
MTC	medullary thyroid carcinoma
US	ultrasound
B-mode	brightness-mode ultrasound
SWE	shear-wave elastography
2D-SWE	two-dimensional shear-wave elastography
ES	elasticity score
ROI	region of interest
FNAC	fine-needle aspiration cytology
TNM	tumor node metastasis staging system
RAI	radioiodine
TSH	thyroid-stimulating hormone
FT4	free thyroxine
Tg	thyroglobulin
anti-Tg	anti-thyroglobulin antibodies
anti-TPO	anti-thyroid peroxidase antibodies
WFUMB	World Federation for Ultrasound in Medicine and Biology
EFSUMB	European Federation of Societies for Ultrasound in Medicine and Biology
